# 2-[(3-Oxo-1-benzofuran-6-yl)­oxy]aceto­nitrile

**DOI:** 10.1107/S1600536812048441

**Published:** 2012-12-12

**Authors:** Henok H. Kinfe, Yonas H. Belay, Zanele H Phasha

**Affiliations:** a Research Center for Synthesis and Catalysis, Department of Chemistry, University of Johannesburg (APK Campus), PO Box 524, Auckland Park, Johannesburg, 2006, South Africa

## Abstract

The mol­ecule of the title compound, C_11_H_8_O_3_, is essentially planar [r.m.s. deviation = 0.025 (2) Å]. In the crystal, mol­ecules are stacked along [110] but no short π–π contacts are observed. Weak C—H⋯O inter­actions link the mol­ecules into chains along [101].

## Related literature

For background to the development of hybrid drug candidates against tuberculosis, malaria and cancer, see: Morphy *et al.* (2004 ▶). For the synthesis of the title compound, see: Hoogendoorn *et al.* (2011 ▶).
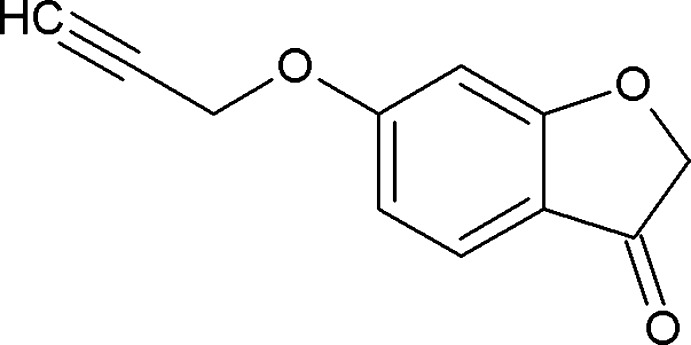


## Experimental

### 

#### Crystal data

C_11_H_8_O_3_
*M* *_r_* = 188.17
Monoclinic, 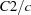

*a* = 16.8785 (5) Å
*b* = 5.4202 (2) Å
*c* = 19.6107 (6) Å
β = 91.469 (2)°
*V* = 1793.49 (10) Å^3^
*Z* = 8
Cu *K*α radiation
μ = 0.85 mm^−1^
*T* = 100 K
0.19 × 0.15 × 0.11 mm

#### Data collection

Bruker APEX DUO 4K CCD diffractometer
Absorption correction: multi-scan (*SADABS*; Bruker, 2008 ▶) *T*_min_ = 0.855, *T*_max_ = 0.912
10685 measured reflections
1545 independent reflections
1451 reflections with *I* > 2σ(*I*)
*R*_int_ = 0.026

#### Refinement

*R*[*F* ^2^ > 2σ(*F* ^2^)] = 0.030
*wR*(*F* ^2^) = 0.076
*S* = 1.04
1545 reflections
127 parameters
H-atom parameters constrained
Δρ_max_ = 0.18 e Å^−3^
Δρ_min_ = −0.13 e Å^−3^

### 

Data collection: *APEX2* (Bruker, 2011 ▶); cell refinement: *SAINT* (Bruker, 2008 ▶); data reduction: *SAINT* and *XPREP* (Bruker, 2008 ▶); program(s) used to solve structure: *SHELXS97* (Sheldrick, 2008 ▶); program(s) used to refine structure: *SHELXL97* (Sheldrick, 2008 ▶); molecular graphics: *ORTEP-3 for Windows* (Farrugia, 2012 ▶); software used to prepare material for publication: *WinGX* (Farrugia, 2012 ▶).

## Supplementary Material

Crystal structure: contains datablock(s) global, I. DOI: 10.1107/S1600536812048441/ld2084sup1.cif

Structure factors: contains datablock(s) I. DOI: 10.1107/S1600536812048441/ld2084Isup2.hkl

Supplementary material file. DOI: 10.1107/S1600536812048441/ld2084Isup3.cml

Additional supplementary materials:  crystallographic information; 3D view; checkCIF report

## Figures and Tables

**Table 1:** Hydrogen-bond geometry (Å, °)

*D*—H⋯*A*	*D*—H	H⋯*A*	*D*⋯*A*	*D*—H⋯*A*
C11—H11⋯O1^i^	0.95	2.24	3.1676 (15)	165

Symmetry code: (i) 
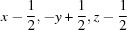
.

**Fig. 1.**
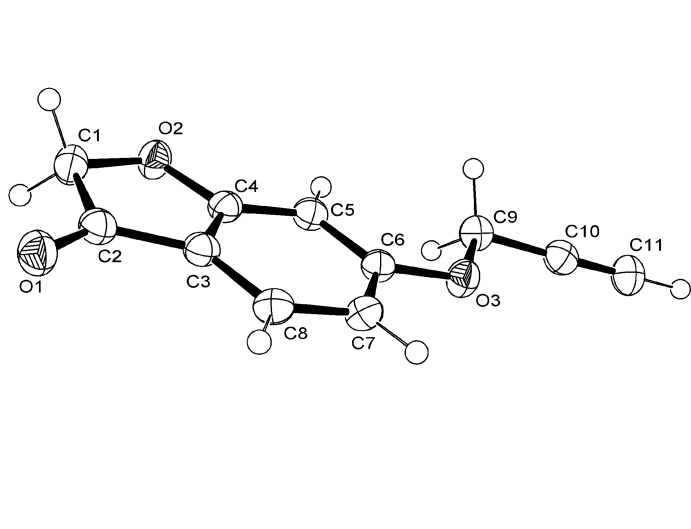
Crystal structure of the title compound with labeling and displacement ellipsoids drawn at the 50% probability level.

**Fig. 2.**
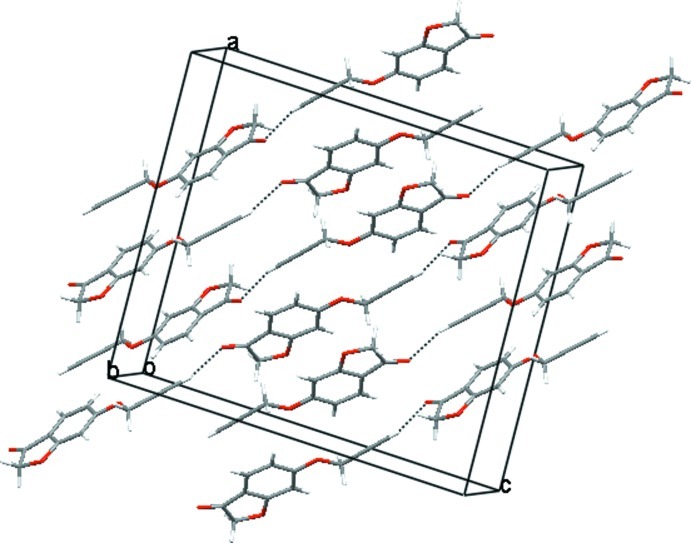
A portion of the crystal packing viewed approximately down the b axis. Dotted lines show intermolecular C–H···O interactions.

## supplementary crystallographic information

### Comment

As a continuation of our progress in the development of hybrid drug candidates against tuberculosis, malaria and cancer (Morphy *et al.*, 2004), the title compound was identified as a promising starting material. The compound was synthesized by reaction of 6–Hydroxy–benzofuran–3–one with propargyl bromide at comparatively low temperature in the presence of potassium carbonate (Hoogendoorn *et al.*, 2011). To confirm the effect of temperature on the reaction, herein we report the single-crystal structure of the title Compound.

The molecular structure of the compound is shown in Figure 1. The molecule is essentially planar (r.m.s. deviation = 0.025 (2) Å). In the crystal the molecules are linked by infinite one-dimensional C–H···O hydrogen bonding into chains that propagate in the [101] direction (Table 1, Figure 2).

### Experimental

A solution of 6–Hydroxy–benzofuran–3–one (1 g, 6.66 mm) in dry acetone was treated with potassium carbonate (1.3 g, 9.32 mm). The reaction mixture was heated at a temperature of 40 – 50 °C for about 30 minutes and then propargyl bromide (1.6 ml, 14.65 mm) was added to it. The combined solution was stirred for about 2.5 h and concentrated under vacuum. The residue was diluted with water and extracted three times with ethyl acetate. The combined organic layer was washed with brine and water and dried over anhydrous magnesium sulfate. After that filtered and the filtrate solid product was recrystalized from ethyl acetate and hexane to afford 80% of the target compound as yellow crystal.

Analytical data: m.p: 112 – 114 oC; ^1^H NMR (CDCl3, 400 MHZ): d 7.56 (d, 1H), 6.69 – 6.64 (m, 2H), 4.74 (s, 2H), 4.60 (s, 2H), 2.57(s, 1H); ^13^C NMR (CDCl3, 400 MHZ): d 197.6, 176.1, 165.8, 125.2, 115.0, 111.9, 97.7, 75.2, 56.2.

### Refinement

All hydrogen atoms were positioned in geometrically idealized positions with C—H = 0.99 Å (methylene), 0.95 Å (aromatic and acetylenic). All hydrogen atoms were allowed to ride on their parent atoms with *U*_iso_(H) = 1.2*U*_eq_. The highest residual electron density of 0.18 e.Å^-3^ is 0.66 Å from C3.

### Crystal data

**Table:**

C_11_H_8_O_3_	*F*(000) = 784
*M**_r_* = 188.17	*D*_x_ = 1.394 Mg m^−^^3^
Monoclinic, *C*2/*c*	Cu *K*α radiation, λ = 1.54178 Å
Hall symbol: -C 2yc	Cell parameters from 6173 reflections
*a* = 16.8785 (5) Å	θ = 6.8–65.7°
*b* = 5.4202 (2) Å	µ = 0.85 mm^−^^1^
*c* = 19.6107 (6) Å	*T* = 100 K
β = 91.469 (2)°	Cube, yellow
*V* = 1793.49 (10) Å^3^	0.19 × 0.15 × 0.11 mm
*Z* = 8	

### Data collection

**Table:**

Bruker APEX DUO 4K CCD diffractometer	1545 independent reflections
Incoatec Quazar Multilayer Mirror monochromator	1451 reflections with *I* > 2σ(*I*)
Detector resolution: 8.4 pixels mm^-1^	*R*_int_ = 0.026
φ and ω scans	θ_max_ = 66.2°, θ_min_ = 6.8°
Absorption correction: multi-scan (*SADABS*; Bruker, 2008)	*h* = −19→18
*T*_min_ = 0.855, *T*_max_ = 0.912	*k* = −6→6
10685 measured reflections	*l* = −22→23

### Refinement

**Table:**

Refinement on *F*^2^	Primary atom site location: structure-invariant direct methods
Least-squares matrix: full	Secondary atom site location: difference Fourier map
*R*[*F*^2^ > 2σ(*F*^2^)] = 0.030	Hydrogen site location: inferred from neighbouring sites
*wR*(*F*^2^) = 0.076	H-atom parameters constrained
*S* = 1.04	*w* = 1/[σ^2^(*F*_o_^2^) + (0.0326*P*)^2^ + 1.2605*P*] where *P* = (*F*_o_^2^ + 2*F*_c_^2^)/3
1545 reflections	(Δ/σ)_max_ < 0.001
127 parameters	Δρ_max_ = 0.18 e Å^−^^3^
0 restraints	Δρ_min_ = −0.13 e Å^−^^3^

### Special details

**Table:**

Experimental. The intensity data was collected on a Bruker Apex DUO 4 K CCD diffractometer using an exposure time of 5 s/frame. A total of 2405 frames were collected with a frame width of 1° covering up to θ = 66.21° with 98.0% completeness accomplished.
Geometry. All s.u.'s (except the s.u. in the dihedral angle between two l.s. planes) are estimated using the full covariance matrix. The cell s.u.'s are taken into account individually in the estimation of s.u.'s in distances, angles and torsion angles; correlations between s.u.'s in cell parameters are only used when they are defined by crystal symmetry. An approximate (isotropic) treatment of cell s.u.'s is used for estimating s.u.'s involving l.s. planes.
Refinement. Refinement of *F*^2^ against ALL reflections. The weighted *R*-factor *wR* and goodness of fit *S* are based on *F*^2^, conventional *R*-factors *R* are based on *F*, with *F* set to zero for negative *F*^2^. The threshold expression of *F*^2^ > 2σ(*F*^2^) is used only for calculating *R*-factors(gt) *etc*. and is not relevant to the choice of reflections for refinement. *R*-factors based on *F*^2^ are statistically about twice as large as those based on *F*, and *R*- factors based on ALL data will be even larger.

### Fractional atomic coordinates and isotropic or equivalent isotropic displacement parameters (Å^2^)

**Table:**

	*x*	*y*	*z*	*U*_iso_*/*U*_eq_	
C1	0.33402 (7)	−0.0134 (2)	0.16647 (6)	0.0301 (3)	
H1A	0.3908	0.0177	0.1582	0.036*	
H1B	0.33	−0.1366	0.2035	0.036*	
C2	0.29260 (7)	0.2247 (2)	0.18581 (6)	0.0275 (3)	
C3	0.22985 (7)	0.2558 (2)	0.13478 (6)	0.0258 (3)	
C4	0.23445 (6)	0.0603 (2)	0.08957 (6)	0.0252 (3)	
C5	0.18383 (6)	0.0313 (2)	0.03363 (6)	0.0256 (3)	
H5	0.188	−0.1042	0.0032	0.031*	
C6	0.12641 (6)	0.2130 (2)	0.02471 (6)	0.0253 (3)	
C7	0.12021 (7)	0.4147 (2)	0.06963 (6)	0.0275 (3)	
H7	0.0802	0.5354	0.0618	0.033*	
C8	0.17159 (7)	0.4373 (2)	0.12444 (6)	0.0277 (3)	
H8A	0.1678	0.5731	0.1548	0.033*	
C9	0.07331 (7)	0.0050 (2)	−0.07344 (6)	0.0281 (3)	
H9A	0.0623	−0.1496	−0.0485	0.034*	
H9B	0.1263	−0.009	−0.0936	0.034*	
C10	0.01328 (7)	0.0443 (2)	−0.12691 (6)	0.0295 (3)	
C11	−0.03534 (7)	0.0700 (2)	−0.17094 (6)	0.0340 (3)	
H11	−0.0744	0.0906	−0.2063	0.041*	
O1	0.31189 (5)	0.35618 (16)	0.23405 (4)	0.0328 (2)	
O2	0.29422 (5)	−0.10199 (15)	0.10488 (4)	0.0296 (2)	
O3	0.07151 (5)	0.21196 (15)	−0.02749 (4)	0.0289 (2)	

### Atomic displacement parameters (Å^2^)

**Table:**

	*U*^11^	*U*^22^	*U*^33^	*U*^12^	*U*^13^	*U*^23^
C1	0.0269 (6)	0.0336 (7)	0.0295 (6)	0.0015 (5)	−0.0041 (5)	0.0012 (5)
C2	0.0270 (6)	0.0289 (6)	0.0265 (6)	−0.0038 (5)	0.0012 (5)	0.0022 (5)
C3	0.0264 (6)	0.0253 (6)	0.0257 (6)	−0.0028 (5)	0.0015 (4)	0.0019 (5)
C4	0.0226 (5)	0.0245 (6)	0.0285 (6)	0.0000 (4)	0.0032 (4)	0.0043 (5)
C5	0.0262 (6)	0.0246 (6)	0.0261 (6)	−0.0008 (5)	0.0029 (4)	−0.0001 (5)
C6	0.0243 (6)	0.0269 (6)	0.0248 (6)	−0.0023 (5)	0.0010 (4)	0.0040 (5)
C7	0.0288 (6)	0.0236 (6)	0.0303 (6)	0.0022 (5)	−0.0001 (5)	0.0025 (5)
C8	0.0313 (6)	0.0232 (6)	0.0288 (6)	−0.0003 (5)	0.0018 (5)	−0.0001 (5)
C9	0.0276 (6)	0.0295 (6)	0.0273 (6)	0.0010 (5)	0.0016 (5)	−0.0016 (5)
C10	0.0279 (6)	0.0311 (6)	0.0296 (6)	−0.0003 (5)	0.0047 (5)	−0.0010 (5)
C11	0.0297 (6)	0.0418 (7)	0.0304 (6)	0.0012 (5)	−0.0018 (5)	−0.0021 (5)
O1	0.0324 (5)	0.0349 (5)	0.0307 (5)	−0.0012 (4)	−0.0048 (3)	−0.0026 (4)
O2	0.0273 (4)	0.0297 (5)	0.0316 (4)	0.0052 (3)	−0.0036 (3)	−0.0019 (3)
O3	0.0288 (4)	0.0298 (4)	0.0278 (4)	0.0034 (3)	−0.0046 (3)	−0.0026 (3)

### Geometric parameters (Å, º)

**Table:**

C1—O2	1.4489 (14)	C6—O3	1.3631 (13)
C1—C2	1.5206 (17)	C6—C7	1.4095 (16)
C1—H1A	0.99	C7—C8	1.3691 (17)
C1—H1B	0.99	C7—H7	0.95
C2—O1	1.2220 (14)	C8—H8A	0.95
C2—C3	1.4481 (16)	C9—O3	1.4396 (14)
C3—C4	1.3851 (16)	C9—C10	1.4551 (16)
C3—C8	1.4021 (17)	C9—H9A	0.99
C4—O2	1.3661 (14)	C9—H9B	0.99
C4—C5	1.3822 (16)	C10—C11	1.1840 (17)
C5—C6	1.3896 (16)	C11—H11	0.95
C5—H5	0.95		
			
O2—C1—C2	106.42 (9)	O3—C6—C7	114.37 (10)
O2—C1—H1A	110.4	C5—C6—C7	122.25 (10)
C2—C1—H1A	110.4	C8—C7—C6	120.30 (11)
O2—C1—H1B	110.4	C8—C7—H7	119.8
C2—C1—H1B	110.4	C6—C7—H7	119.8
H1A—C1—H1B	108.6	C7—C8—C3	118.58 (11)
O1—C2—C3	129.99 (11)	C7—C8—H8A	120.7
O1—C2—C1	124.96 (11)	C3—C8—H8A	120.7
C3—C2—C1	105.04 (9)	O3—C9—C10	108.15 (9)
C4—C3—C8	119.66 (10)	O3—C9—H9A	110.1
C4—C3—C2	107.53 (10)	C10—C9—H9A	110.1
C8—C3—C2	132.81 (11)	O3—C9—H9B	110.1
O2—C4—C5	122.62 (10)	C10—C9—H9B	110.1
O2—C4—C3	113.91 (10)	H9A—C9—H9B	108.4
C5—C4—C3	123.47 (11)	C11—C10—C9	178.27 (13)
C4—C5—C6	115.74 (11)	C10—C11—H11	180
C4—C5—H5	122.1	C4—O2—C1	107.09 (9)
C6—C5—H5	122.1	C6—O3—C9	116.64 (9)
O3—C6—C5	123.37 (10)		
			
O2—C1—C2—O1	−177.45 (10)	C4—C5—C6—C7	0.20 (16)
O2—C1—C2—C3	1.34 (12)	O3—C6—C7—C8	179.74 (10)
O1—C2—C3—C4	177.78 (12)	C5—C6—C7—C8	−0.07 (17)
C1—C2—C3—C4	−0.93 (12)	C6—C7—C8—C3	−0.23 (17)
O1—C2—C3—C8	−1.2 (2)	C4—C3—C8—C7	0.40 (16)
C1—C2—C3—C8	−179.91 (12)	C2—C3—C8—C7	179.28 (11)
C8—C3—C4—O2	179.30 (10)	C5—C4—O2—C1	−179.70 (10)
C2—C3—C4—O2	0.16 (13)	C3—C4—O2—C1	0.73 (12)
C8—C3—C4—C5	−0.27 (17)	C2—C1—O2—C4	−1.26 (12)
C2—C3—C4—C5	−179.41 (10)	C5—C6—O3—C9	2.07 (15)
O2—C4—C5—C6	−179.56 (10)	C7—C6—O3—C9	−177.74 (9)
C3—C4—C5—C6	−0.03 (16)	C10—C9—O3—C6	−177.25 (9)
C4—C5—C6—O3	−179.59 (10)		

### Hydrogen-bond geometry (Å, º)

**Table:**

*D*—H···*A*	*D*—H	H···*A*	*D*···*A*	*D*—H···*A*
C11—H11···O1^i^	0.95	2.24	3.1676 (15)	165

Symmetry code: (i) *x*−1/2, −*y*+1/2, *z*−1/2.
